# Soluble receptor for advanced glycation end products protects from ischemia- and reperfusion-induced acute kidney injury

**DOI:** 10.1242/bio.058852

**Published:** 2022-02-04

**Authors:** Taro Miyagawa, Yasunori Iwata, Megumi Oshima, Hisayuki Ogura, Koichi Sato, Shiori Nakagawa, Yuta Yamamura, Yasutaka Kamikawa, Taito Miyake, Shinji Kitajima, Tadashi Toyama, Akinori Hara, Norihiko Sakai, Miho Shimizu, Kengo Furuichi, Seiichi Munesue, Yasuhiko Yamamoto, Shuichi Kaneko, Takashi Wada

**Affiliations:** 1 Department of Nephrology and Laboratory Medicine, Kanazawa University, 13-1 Takara-machi, Kanazawa 920-8641, Japan; 2 Division of Infection Control, Kanazawa University Hospital, 13-1 Takara-machi, Kanazawa 920-8641, Japan; 3 Division of Blood Purification, Kanazawa University Hospital, 13-1 Takara-machi, Kanazawa 920-8641, Japan; 4 Department of Nephrology, Kanazawa Medical University School of Medicine, 1-1 Daigaku, Uchinada, Kahoku, Ishikawa 920-0293, Japan; 5 Department of Biochemistry and Molecular Vascular Biology, Kanazawa University Graduate School of Medical Sciences, 13-1 Takara-machi, Kanazawa 920-8641, Japan; 6 Department of System Biology, Institute of Medical Pharmaceutical and Health Science, Kanazawa University, 13-1 Takara-machi, Kanazawa 920-8641, Japan

**Keywords:** Receptor for advanced glycation end products (RAGE), Soluble receptor for advanced glycation end products (sRAGE), Ischemia and reperfusion, Acute kidney injury, High-mobility group box 1 (HMGB1)

## Abstract

The full-length receptor for advanced glycation end products (RAGE) is a multiligand pattern recognition receptor. High-mobility group box 1 (HMGB1) is a RAGE ligand of damage-associated molecular patterns that elicits inflammatory reactions. The shedded isoform of RAGE and endogenous secretory RAGE (esRAGE), a splice variant, are soluble isoforms (sRAGE) that act as organ-protective decoys. However, the pathophysiologic roles of RAGE/sRAGE in acute kidney injury (AKI) remain unclear. We found that AKI was more severe, with enhanced renal tubular damage, macrophage infiltration, and fibrosis, in mice lacking both RAGE and sRAGE than in wild-type (WT) control mice. Using murine tubular epithelial cells (TECs), we demonstrated that hypoxia upregulated messenger RNA (mRNA) expression of HMGB1 and tumor necrosis factor α (TNF-α), whereas RAGE and esRAGE expressions were paradoxically decreased. Moreover, the addition of recombinant sRAGE canceled hypoxia-induced inflammation and promoted cell viability in cultured TECs. sRAGE administration prevented renal tubular damage in models of ischemia/reperfusion-induced AKI and of anti-glomerular basement membrane (anti-GBM) glomerulonephritis. These results suggest that sRAGE is a novel therapeutic option for AKI.

## Abstract (precis)

**Summary:** Soluble (s) RAGE reduced tubular injury in mice acute kidney injury (AKI) model. sRAGE attenuated hypoxia induced damage and accelerated proliferation in tubular epithelial cells. sRAGE may be a potential therapeutic option for AKI.

## INTRODUCTION

Acute kidney injury (AKI) is one of the important risk factors for the development of chronic kidney disease and end-stage renal disease (ESRD) (Basile et al., 2016). Renal ischemia and reperfusion (I/R), which leads to AKI, occurs in humans in settings such as trauma, circulatory arrest, major vascular surgery, and kidney transplantation (Eltzschig and Eckle, 2011). Renal I/R induces immune responses through inflammatory signaling transductions. Pattern recognition receptors, such as Toll-like receptors, recognize pathogen-related molecules and activate an inflammatory response. Recent studies have revealed that Toll-like receptors play a critical role even in I/R-induced sterile inflammation (Eltzschig and Eckle, 2011; Wu et al., 2010).

A full-length form of the receptor for advanced glycation end products (RAGE) is also a multiligand pattern recognition receptor (Bongarzone et al., 2017). RAGE binds advanced glycation end products, S100 proteins, high-mobility group box protein 1 (HMGB1), and β-sheet fibrillar material (Bongarzone et al., 2017; Schmidt et al., 2001; Sims et al., 2010). HMGB1, one of the damage-associated molecular patterns (DAMPs), is a common ligand for RAGE and Toll-like receptors. The HMGB1–RAGE association activates pro-inflammatory signal transduction via nuclear factor κ-light chain-enhancer of activated B cells, resulting in inflammatory responses (Batkulwar et al., 2015; Chen et al., 2016). It is known that soluble isoforms of RAGE (sRAGE) consist of cleaved isoforms of RAGE and endogenous secretory RAGE (esRAGE) (Prasad, 2019). Cleaved RAGE is derived by the proteolytic cleavage of full-length RAGE, whereas esRAGE is generated by alternative splicing of RAGE messenger RNA (mRNA) (Prasad, 2019; Yonekura et al., 2003; Kalea et al., 2009; Schmidt, 2015). RAGE has two ways of affecting DAMP signaling. The full-length signal-transducing RAGE binds DAMPs, eliciting inflammatory reactions; sRAGE captures DAMP-related ligands and inhibits the intracellular signal transductions as a decoy-type receptor (Bongarzone et al., 2017). sRAGE plays an important role in protecting organs in various pathologic conditions, such as acute lung injury, diabetic atherosclerosis, Alzheimer's disease, and septic shock (Park et al., 1998; Yamamoto et al., 2011; Sugihara et al., 2012; Blondonnet et al., 2017).

Although the relationship between RAGE and the pathophysiologic features of various kidney diseases, such as chronic unilateral ureteral obstruction and autosomal dominant polycystic kidney disease, has been reported, the effect of RAGE/sRAGE on AKI remains unclear (Gasparitsch et al., 2013; Lee et al., 2015). We therefore explored the pathophysiologic role of RAGE and sRAGE in a mouse model of AKI.

## RESULTS

### RAGE or sRAGE is involved in renal tubular damage in murine I/R-induced AKI model

**Fig. 1.**
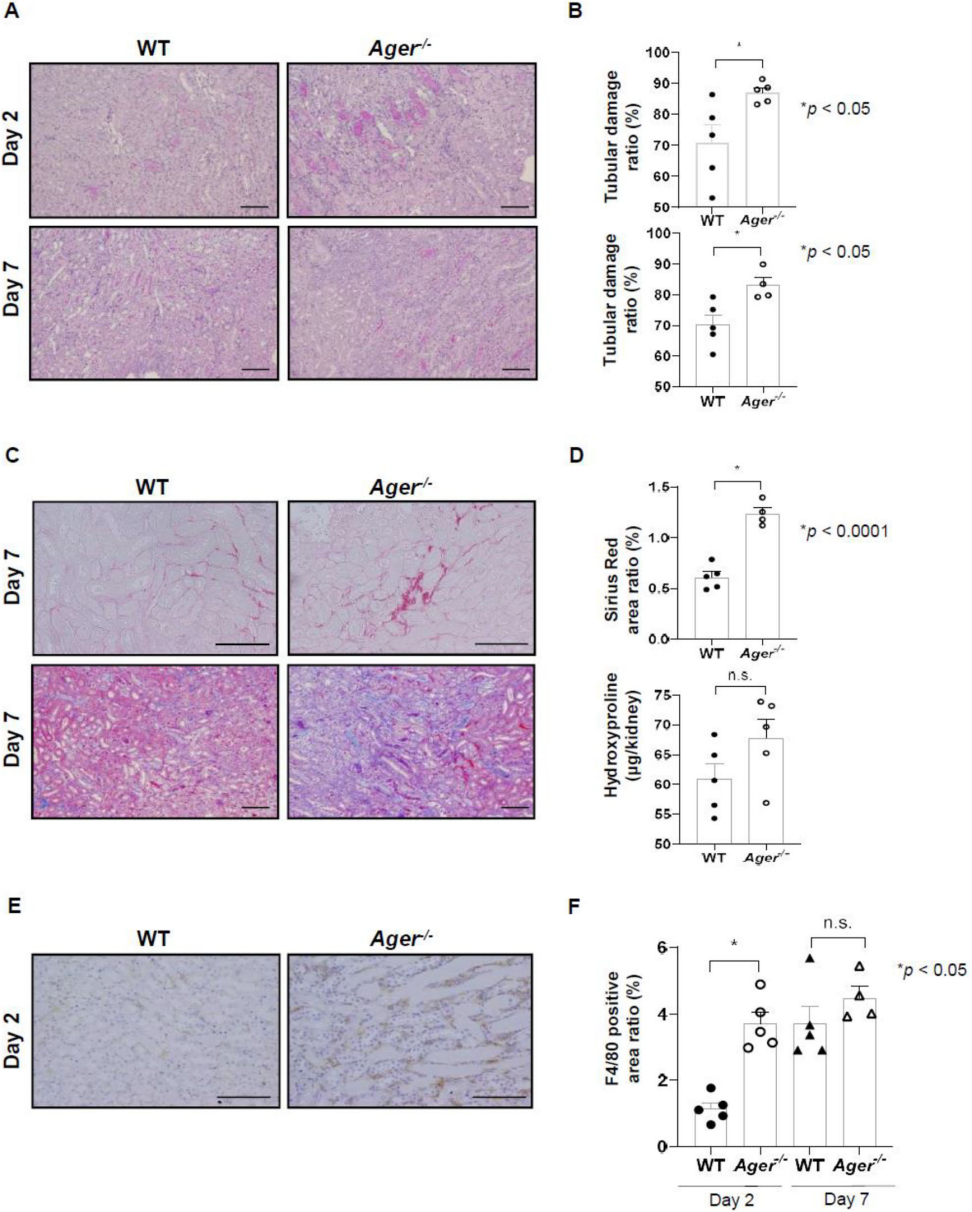
**Receptor for advanced glycation end products and soluble receptor for advanced glycation end products were involved in renal tubular damage in a murine model of ischemia and reperfusion (I/R)-induced acute kidney injury.** (A) Representative images of tissue samples with periodic acid–Schiff staining of the corticomedullary junction (days 2 and 7; magnification, ×100). Scale bars: 100 µm. (B) Tubular damage was more severe in *Ager^−/−^* mice than in the control WT mice 2 and 7 days after I/R injury (day 2, 5 mice; day 7, 5 WT mice and 4 *Ager^−/−^* mice). (C) Representative images of tissue samples with Sirius red staining (upper, day 7; magnification, ×200) and Azan staining (lower, day 7; magnification, ×100) of the corticomedullary junction. Scale bars: 100 µm. (D) The percentage of the area positive for Sirius red staining was greater in the *Ager^−/−^* mice than in the control mice 7 days after I/R injury (5 WT and 4 *Ager^−/−^* mice), although hydroxyproline levels were not significantly increased (5 mice). (E) Representative images of tissue samples with F4/80 staining of the corticomedullary junction 2 days after I/R injury (magnification, ×200). Scale bars: 100 µm. (F) The percentage of the area positive for F4/80 staining was greater in the *Ager^−/−^* mice than in the WT mice after I/R injury (day 2, 5 mice; day 7, 5 WT and 4 *Ager^−/−^* mice). Data were expressed as means±s.e.m.

To assess whether RAGE or sRAGE was involved in renal tubular damage, we induced unilateral renal I/R injury both in *Ager^−/−^* mice and WT control mice. Tubular damage, interstitial cell accumulation, and fibrosis were analyzed 2 and 7 days after I/R injury. Tubular damage, which consisted of cast formation, tubular necrosis, loss of the brush border, and tubular dilatation, was more severe in *Ager^−/−^* mice than in control mice 2 and 7 days after I/R injury (Fig. 1A,B; *P*<0.05). Sirius Red staining showed that kidney fibrosis was more severe in *Ager^−/−^* mice than in the control mice on day 7, although hydroxyproline levels were not significantly increased (Fig. 1C,D; *P*<0.0001). Furthermore, significant macrophage infiltration was also exaggerated in *Ager^−/−^* mice in comparison with the control mice on day 2 (Fig. 1E,F; *P*<0.05).

### Downregulation of full-length RAGE and esRAGE mRNA expressions by hypoxic stimulation

**Fig. 2.**
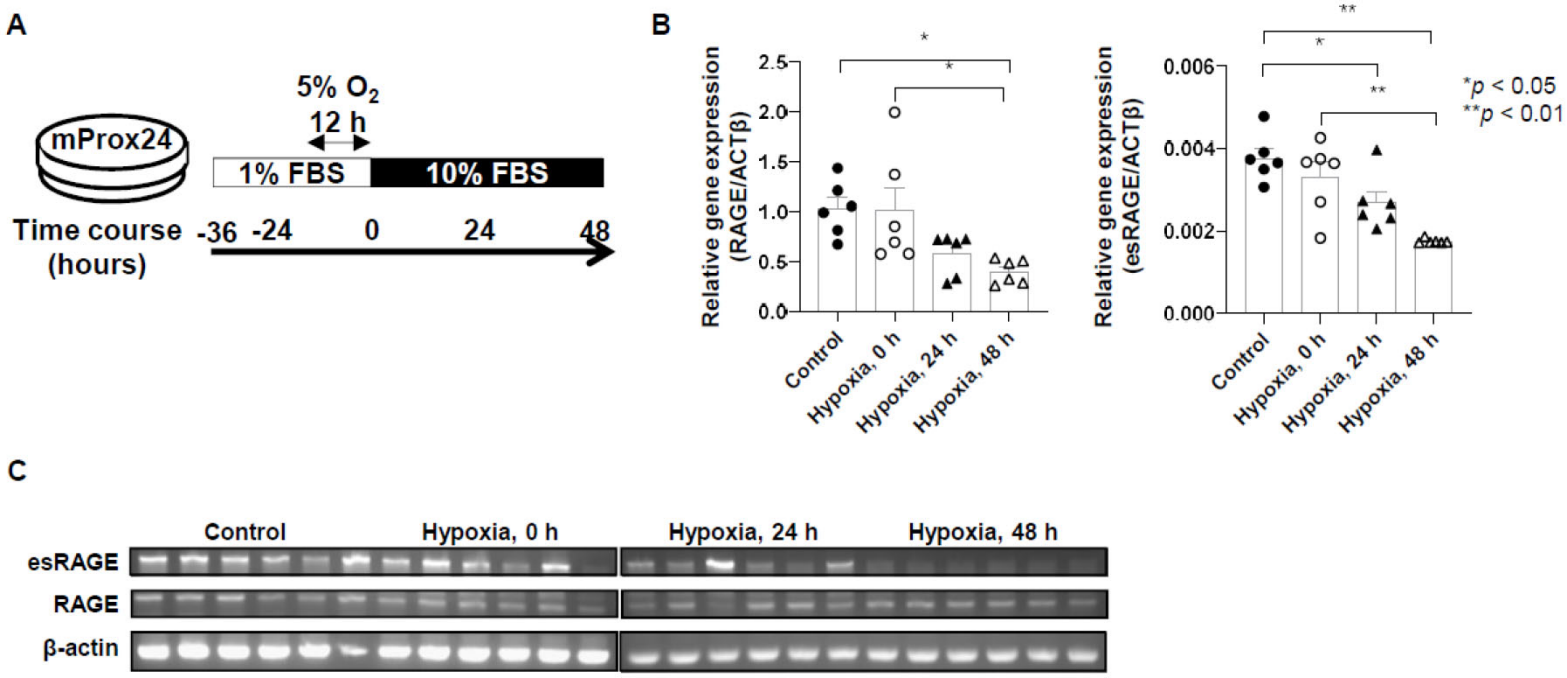
**Downregulation of full-length RAGE and esRAGE messenger RNA expressions by hypoxic stimulation.** (A) Protocol for the induction of hypoxia. (B,C) The induction of hypoxia decreased the expression of genes coding full-length RAGE and esRAGE in murine renal proximal tubular epithelial cells over time (6 cells from each group). Data on the left are expressed as copies of full-length RAGE mRNA relative to copies of β-actin mRNA in quantitative real-time polymerase chain reaction (PCR). Data on the right are expressed as mean density of esRAGE bands relative to β-actin bands in semiquantitative PCR. Data were expressed as means±standard errors of the mean. ACTβ, β-actin; FBS, fetal bovine serum.

To examine the expression level of each RAGE isoform in damaged tubular epithelial cells (TECs), we analyzed the gene expression of full-length RAGE and esRAGE in murine renal proximal tubular epithelial (mProx24) cells subjected to hypoxia (Fig. 2A). Hypoxia decreased the expression of genes coding full-length RAGE and esRAGE in mProx24 cells over time (Fig. 2B,C; *P*<0.05).

### Treatment of sRAGE downregulates pro-inflammatory mediators and induces proliferation of mProx24 cells subjected to hypoxia

**Fig. 3.**
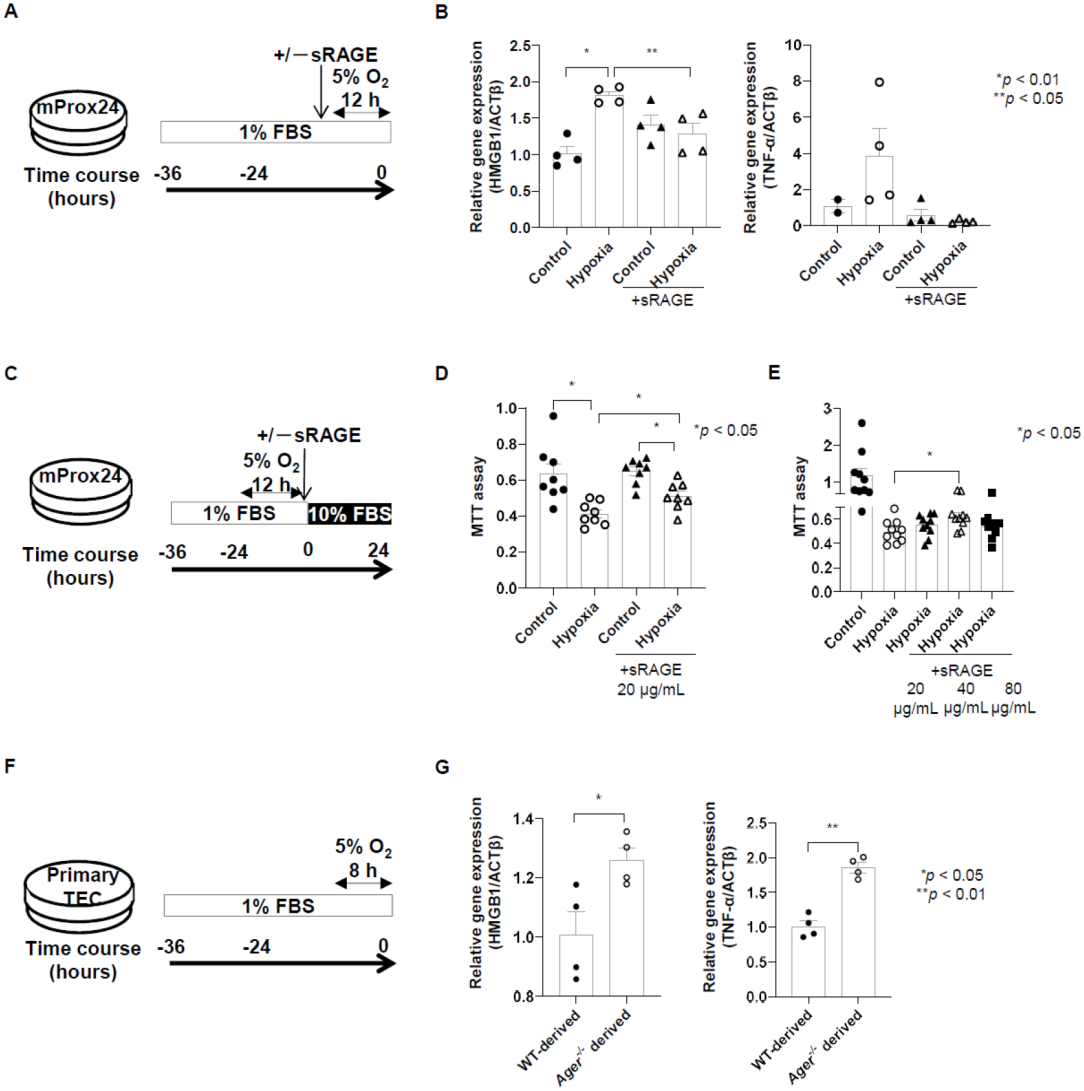
**Treatment with soluble receptor for sRAGE downregulated pro-inflammatory mediators and induced the proliferation in murine renal proximal tubular epithelial (mProx24) cells subjected to hypoxia.** (A) Protocol for the induction of hypoxia with the addition of sRAGE for mProx24 cells. (B) The induction of hypoxia upregulated high-mobility group box 1 (HMGB1) and tumor necrosis factor α (TNF-α) mRNA in mProx24 cells. However, the addition of sRAGE decreased *Hmbg1* and *Tnfa* mRNA in hypoxic mProx24 cells (*Hmgb1*, 4 cells from each group; *Tnfa*, 2 cells from the control group, four from the other groups). (C) Protocol for MTT assay with the addition of sRAGE for mProx24 cells. (D,E) The addition of sRAGE induced the proliferation of hypoxic mProx24 cells in a dose-dependent manner (D: 8 cells from each group; E: 1 cell from each group). (F) Protocol for studying primary tubular epithelial cells (TECs). (G) The expression of HMGB1 and TNF-α mRNA was greater in primary TECs in *Ager^−/−^* mice after hypoxia than in the control WT mice (4 cells from each group). Data were expressed as means±standard errors of the mean. FBS, fetal bovine serum.

We then evaluated the expression of genes coding for pro-inflammatory mediators after hypoxia was induced in mProx24 cells (Fig. 3A). Hypoxia caused mRNA upregulation of *Hmgb1* and *Tnfa* in mProx24 cells (Fig. 3B). However, the addition of sRAGE decreased *Hmgb1* and *Tnfa* mRNA levels in hypoxic mProx24 cells (Fig. 3B). We also assessed cellular damage in hypoxic mProx24 cells with or without the addition of sRAGE (Fig. 3C). The sRAGE induced proliferation of hypoxic mProx24 cells (Fig. 3D; *P*<0.05). The addition of sRAGE induced the proliferation of TECs after their subjection to hypoxia and was most effective at 40 µg/ml (Fig. 3E; *P*<0.05). Moreover, we assessed the expression of genes coding for pro-inflammatory mediators after hypoxia in primary TECs (Fig. 3F). *Hmgb1* and *Tnfa* mRNA were expressed more in primary TEC-derived *Ager^−/−^* mice after hypoxia than in the control mice (Fig. 3G; *P*<0.05).

### Administration of sRAGE protects renal tubules from ischemic reperfusion injury

**Fig. 4.**
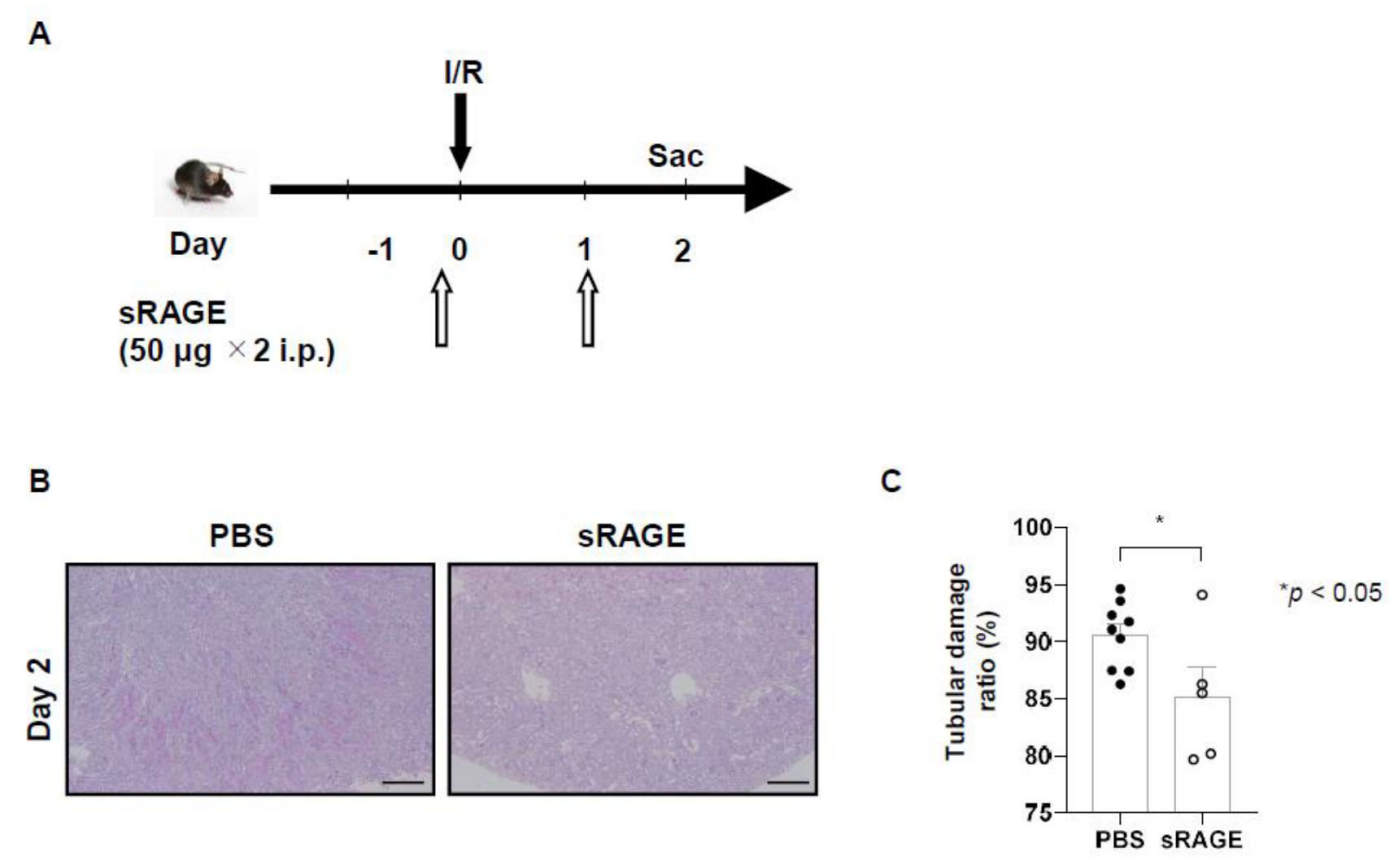
**The administration of sRAGE protects renal tubules from ischemic reperfusion injury.** (A) Protocol for the administration of sRAGE to mice with ischemia and reperfusion (I/R) induced acute kidney injury. (B) Representative images of tissue samples with periodic acid–Schiff staining of the corticomedullary junction 2 days after I/R injury (magnification, ×100). Scale bars: 100 µm. (C) Two days after I/R injury in B6 mice, sRAGE administration (in 9 mice) reduced tubular damage more than did phosphate-buffered saline (in 5 mice). Data were expressed as means±standard errors of the mean. i.p., intraperitoneally; Sac, sacrifice.

We then explored the renoprotective effects of sRAGE *in vivo*. Unilateral renal I/R was induced in WT mice with or without sRAGE administration. Tubular damage was assessed 2 days later (Fig. 4A). Among the B6 mice subjected to I/R, sRAGE administration reduced tubular damage more than that caused by phosphate-buffered saline (PBS; Fig. 4B,C; *P*<0.05).

### Administration of sRAGE administration also protects from tubular damage caused by anti-glomerular basement membrane glomerulonephritis

**Fig. 5.**
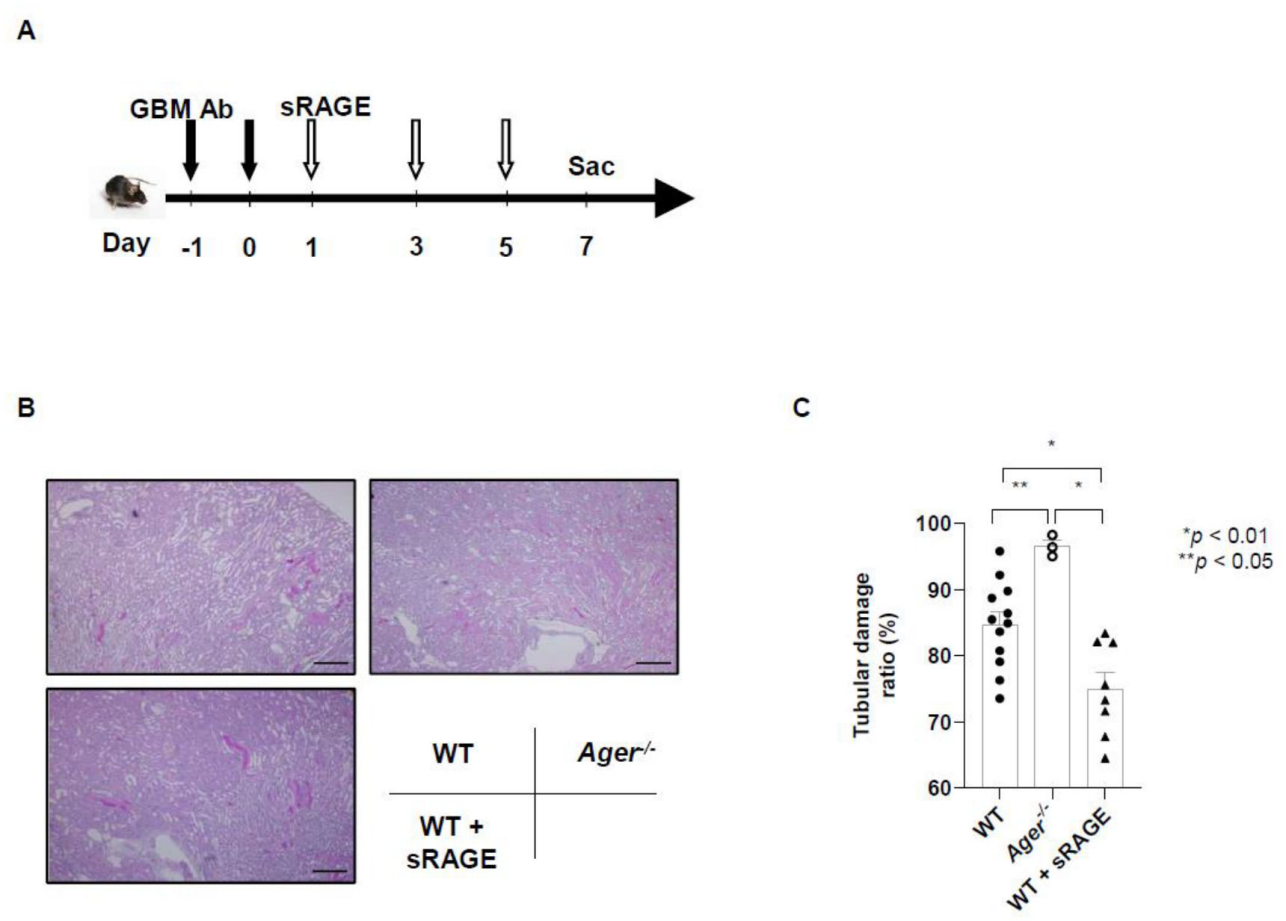
**The administration of sRAGE also protects from tubular damage of anti-glomerular basement membrane (anti-GBM) glomerulonephritis.** (A) Protocol for the administration of sRAGE to mice with anti-GBM glomerulonephritis. (B) Representative images of tissue samples with periodic acid–Schiff staining of the corticomedullary junction 7 days after the induction of anti-GBM glomerulonephritis (magnification, ×100). Scale bars: 100 µm. (C) Tubular damage was more severe in *Ager^−/−^* mice than in WT mice. The administration of sRAGE reduced the severity of tubular damage than in the other two groups 7 days after the induction of anti-GBM glomerulonephritis (12 WT mice, 3 *Ager^−/−^* mice, and 8 WT mice that received sRAGE). Data were expressed as means±standard errors of the means. Ab, antibody; Sac, sacrifice.

Next, to determine whether sRAGE has therapeutic potential in another model, we assessed tubular damage from anti-glomerular basement membrane (anti-GBM) glomerulonephritis in mice on day 7, in accordance with the protocol that comprised of preparing anti-GBM glomerulonephritis model mice, administering sRAGE, and then sacrificing them on day 7. (Fig. 5A). Kidneys from WT mice that received sRAGE and from *Ager^−/−^* mice were evaluated with the injection of anti-GBM antibody. Tubular damage was more severe in *Ager^−/−^* mice than in WT mice. In mice that received sRAGE, tubular damage was less severe than in the other two groups (Fig. 5B,C).

## DISCUSSION

This study aims at exploring the pathophysiologic role of RAGE and sRAGE in tubular injury *in vivo* and *in vitro*. In an AKI model, we demonstrated that renal tubular damage was more severe in *Ager^−/−^* mice than in control mice. The expression of full-length RAGE and esRAGE mRNA was downregulated in TECs under conditions of hypoxia. Furthermore, the administration of sRAGE caused downregulation of pro-inflammatory mediators and induced proliferation of hypoxic TECs. Finally, sRAGE protected against renal tubular damage in models of both AKI and anti-GBM glomerulonephritis.

Recent studies have revealed that RAGE is involved in the pathogenesis of I/R-induced injury to the lungs (Sharma et al., 2013), heart (Wang et al., 2018), and brain (Liu et al., 2020). However, few studies have focused on the pathophysiologic roles of RAGE in AKI. We demonstrated that renal tubular damage was exacerbated in *Ager^−/−^* mice subjected to I/R in comparison with control mice and was less severe in WT mice subjected to I/R and sRAGE administration. In support of our hypothesis, sRAGE reportedly acted as a decoy in inhibiting RAGE-related inflammatory signal transduction (Bongarzone et al., 2017). Moreover, sRAGE expression was abundant in mouse kidneys, although the expression levels of isoforms of RAGE differed according to the species and organs (Harashima et al., 2006; Jules et al., 2013).

RAGE was reported to be upregulated in various types of chronic kidney disease, such as diabetic nephropathy, hypertensive nephropathy, and obesity-related glomerulopathy (D'Agati and Schmidt, 2010). The serum levels of sRAGE are also known to increase in patients with ESRD (Kalousova et al., 2006). We demonstrated that the expression of full-length RAGE and esRAGE mRNA was downregulated by hypoxia induced in mProx24 cells over time. This result suggests that acute hypoxia induces downregulation of RAGE and sRAGE in the renal tubules, which results in failure to inhibit inflammatory signal transductions and may exacerbate renal tubular damage. However, the molecular mechanisms of RAGE and sRAGE expression remain unclear in TECs subjected to hypoxia.

Hypoxia induction was reported to cause upregulation of pro-inflammatory mediators, such as HMGB1, TNF-α, and monocyte chemotactic protein-1 in the kidneys and human leukocytes (Bai et al., 2018; Zhang et al., 2019). We obtained similar results in hypoxic mProx24 cells, and the addition of sRAGE downregulated the mRNA expression of pro-inflammatory mediators. Furthermore, the induction of hypoxia has been shown to inhibit the growth of rat TECs (Zhu et al., 2018). We found that cell proliferation was also inhibited in hypoxic mProx24 cells; moreover, cell proliferation was restored by the addition of sRAGE. This result suggests that sRAGE may reduce the inflammation and enhance the proliferation of TECs in conditions of hypoxia. In addition, the expression of pro-inflammatory mediators was enhanced in *Ager^−/−^*-derived primary TECs in comparison with WT-derived primary TECs. These findings would support our hypothesis that sRAGE may protectively act against I/R-induced AKI.

We showed that sRAGE treatment also reduced renal damage in a mouse model of AKI. In support of our hypothesis, sRAGE administration has been reported to reduce acute organ damage, such as acute lung injury, and sepsis (Yamamoto et al., 2011; Blondonnet et al., 2017). Moreover, it is known that anti-GBM glomerulonephritis causes not only glomerular damage, but also tubular damage (Andres et al., 1978). We confirmed the therapeutic effect of sRAGE in models other than tubular damage caused by I/R injury. These results indicated that sRAGE has protective roles in tubular injury, regardless of the underlying cause of tubular damage. Thus, sRAGE could be a promising therapeutic option for kidney injury.

This study had several limitations. First, the molecular mechanisms involved in RAGE and AKI caused by I/R have not been fully evaluated. Because HMGB1 is involved even in aseptic inflammation, such as that caused by I/R, we assume that the HMGB1–RAGE signal was responsible for kidney injury in our model. However, we did not analyze the interaction between RAGE and ligands such as HMGB1. Moreover, the roles of other pattern recognition receptors, such as Toll-like receptors, need to be elucidated.

**Fig. 6.**
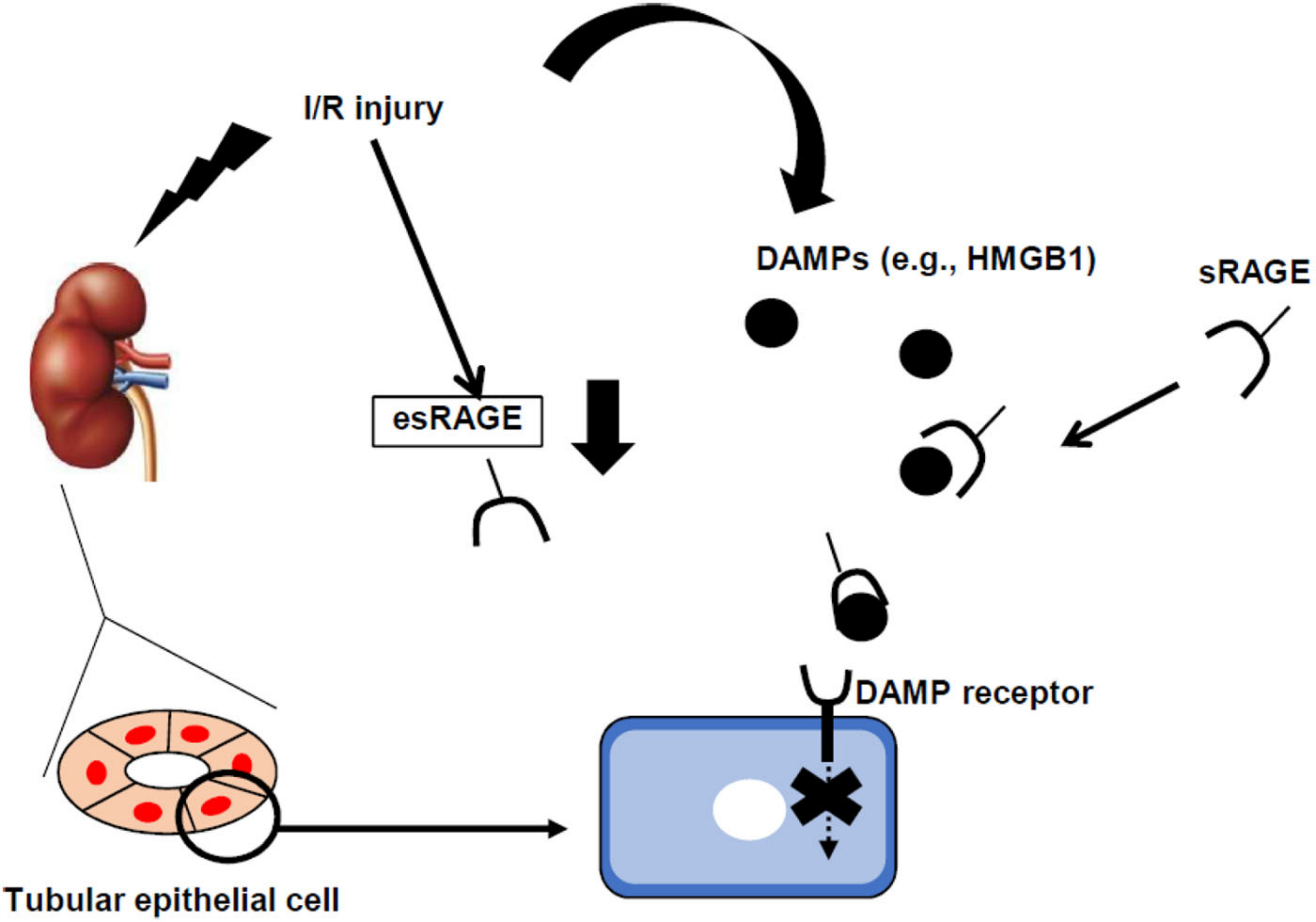
**Proposed diagram of the relationship between full-length RAGE, sRAGE, and ischemia and reperfusion-induced acute kidney injury, according to the results of this study.** DAMPs, damage-associated molecular patterns; esRAGE, endogenous secretory RAGE; HMGB1, high-mobility group box 1.

In conclusion, we have shown the protective role of sRAGE in renal I/R-induced tubular damage (Fig. 6). Hypoxic stimulation downregulated the expression of full-length RAGE/esRAGE in TECs, which in turn might reduce the capture ability of DAMPs, such as HMGB1. These uncaptured DAMPs would have exacerbated tubular injury in our model. Hence, sRAGE administration showed a renoprotective effect in tubular injury. These findings help clarify molecular mechanisms and indicate novel therapeutic options for AKI.

## MATERIALS AND METHODS

### Animals

We purchased male C57BL/6J (WT) mice from Charles River Japan (Yokohama, Japan). RAGE-deficient [*Ager* knockout (*Ager^−/−^*); C57BL/6J background] mice were produced by crossbreeding heterozygous mutant mice (Myint et al., 2006). The C57BL/6J and *Ager^−/−^* mice were housed and bred at Kanazawa University, Kanazawa, Japan. All animal experiments were conducted in accordance with the guidelines for animal care of Kanazawa University and were approved by the Institute for Experimental Animals, Kanazawa University Advanced Science Research Center (registration number, AP-153391). This study is reported following the recommendations of the ARRIVE guidelines (https://arriveguidelines.org).

### Renal I/R injury model

We induced kidney I/R injury as previously described (Iwata et al., 2012). To induce ischemia, we clamped the left renal pedicle with a nontraumatic clip (Natsume Seisakusho, Tokyo, Japan) after the induction of anesthesia. The clip was removed after 50 min. To control body temperature, we placed the mice on a 37.0°C heating pad throughout the procedure.

### Preparation of anti-mouse glomerular basement membrane antibodies

We prepared mouse glomerulus according to the method of Krakower and Greenspon (Krakower and Greenspon, 1951) and anti-mouse GBM antibodies as previously described (Wada et al., 1996). To confirm specificity, we performed *in vitro* indirect immunofluorescence using fluorescein isothiocyanate-conjugated anti-rabbit immunoglobulin G (Organon Teknika Corporation, Durham, NC, USA) on frozen sections of normal mouse kidneys. Sharp linear immunofluorescence was obtained along the GBM.

### Anti-GBM glomerulonephritis model

We induced anti-GBM glomerulonephritis in mice as previously described (Wada et al., 1996) with some modifications. In both WT and *Ager^−/−^* mice, 0.5 ml of nephrotoxic serum was intraperitoneally injected on days –1 and 0. Each mouse was sacrificed on day 7. The mice with anti-GBM glomerulonephritis were divided into three groups: the WT group, in which WT mice received vehicle PBS by intraperitoneal injection on days 1, 3, and 5; the *Ager^−/−^* group, in which *Ager^−/−^* mice received vehicle PBS by intraperitoneal injection on days 1, 3, and 5; and the WT+sRAGE group, in which WT mice received recombinant sRAGE (50 μg each time) by intraperitoneal injection on days 1, 3, and 5.

### Reagents

Recombinant mouse soluble RAGE was provided by Yasuhiko Yamamoto (Kanazawa University, Kanazawa, Japan) (Yamamoto et al., 2011).

### Murine tubular epithelial cell line

The murine TEC line mProx24 was provided by Takeshi Sugaya (St. Marianna University School of Medicine, Tokyo, Japan). In each *in vitro* experiment, the cells were cultured in Dulbecco's modified Eagle's medium (DMEM; Thermo Fisher Scientific, Waltham, MA, USA) with 10% fetal bovine serum (FBS) and 1% penicillin/streptomycin for cell culture and in DMEM with 1% FBS after a 24-h period of serum starvation.

### Primary culture of murine renal TECs

Primary murine renal TECs from WT and *Ager^−/−^* mice were generated in accordance with the method described in Wuthrich et al. (1990), with some modifications. The kidneys were stripped of blood cells by being washed in saline. The kidney cortices from WT and *Ager^−/−^* mice were cut into small pieces and then digested in defined K1 medium containing 4 mg/ml of collagenase at 37°C for 1 h. The digested kidney pieces were washed with a cell strainer (mesh diameters of 100 and 40 μm). The cortical tubular cells were spun down at 1500 rpm for 5 min and washed again. The cell pellet was resuspended in defined K1 medium. The cell suspension was placed on cell culture Petri dishes and incubated at 37°C. The experiments were performed after the cells had reached 80%–90% confluence. Primary TECs were stimulated in serum-free K1 medium for 10 h starvation and then placed in a chamber with 5% O_2_ for 8 h according to the protocol (Fig. 3F).

### Renal histopathology

A sample of kidneys from each mouse was fixed in 10% buffered formalin (pH 7.2) and embedded in paraffin. We stained 5-μm sections with periodic acid–Schiff and Azan reagents. The percentage of proximal tubules at the corticomedullary junction that displayed proximal tubule dilation, a loss of the brush border, the presence of casts, and cellular necrosis were counted. The specimens were evaluated in a blinded manner from at least 10 different kidney sections (magnification, ×200) for each sample. Staining with F4/80 (catalog no. MF48000; Invitrogen, Carlsbad, CA, USA) and Sirius Red stain was performed as previously described (Iwata et al., 2012).

### RNA analyses

To isolate total cellular RNA from the cultured cells, we used the High Pure RNA Isolation Kit (Roche Diagnostics K. K., Tokyo, Japan) and the ISOSPIN cell and tissue RNA (NIPPON Gene, Tokyo, Japan). We performed quantitative real-time polymerase chain reaction (PCR) analysis with iQ™ SYBR^®^ Green Supermix (catalog no. 170-8885; BioRad, Hercules, CA, USA) using the ViiA™7 Real-Time PCR System (Thermo Fisher Scientific). The following primers were used: *Ager* (catalog no. Mm_Ager_1_SG, QuantiTect Primer Assay; Qiagen, Hilden, Germany), HMGB1 (catalog no. Mm00849805_gH, TaqMan Gen Expression Assay; Applied Biosystems, Foster City, CA, USA), *Tnfa* (catalog no. Mm_TNF_1_SG, QuantiTect Primer Assay; Qiagen), and *Actb* (catalog no. 4352341E, Mouse ACTB Endogenous Control; Applied Biosystems). Data were analyzed according to the delta-delta Ct method (Iwata et al., 2017). Semiquantitative PCR for full-length RAGE, esRAGE, and β-actin were performed. The PCR products were analyzed by electrophoresis on 2% agarose in 1× TAE buffer (100 V, 20 min). Data were analyzed using the ImageJ software (https://imagej.nih.gov/ij/).

### Cell proliferation assay

TEC proliferation was determined using the Cell Counting Kit-8 (catalog no. CK04; Dojindo, Kumamoto, Japan) in accordance with the manufacturer's instructions, as previously described (Iwata et al. 2010).

### Hydroxyproline assay

I/R-injured kidneys were taken from each mouse to assess the amount of kidney collagen. The hydroxyproline assay ware performed according to the standard protocol of our laboratory, as previously described (Sakai et al., 2013). Assay results were expressed as micrograms of hydroxyproline per kidney.

### Statistical analysis

The data are expressed as means±s.e.m. To perform the statistical analysis, we used the two-tailed unpaired student's *t*-test to compare the two groups; we also used one-way analysis of variance with Tukey's multiple comparison test to compare more than two groups (GraphPad Prism 8 software). *P*-values <0.05 were considered statistically significant.

## References

[BIO058852C1] Andres, G., Brentjens, J., Kohli, R., Anthone, R., Anthone, S., Baliah, T., Montes, M., Mookerjee, B. K., Prezyna, A., Sepulveda, M. et al. (1978). Histology of human tubulo-interstitial nephritis associated with antibodies to renal basement membranes. *Kidney Int.* 13, 480-491. 10.1038/ki.1978.71 362036

[BIO058852C2] Bai, W., Zhou, J., Zhou, N., Liu, Q., Cui, J., Zou, W. and Zhang, W. (2018). Hypoxia-increased RAGE expression regulates chemotaxis and pro-inflammatory cytokines release through nuclear translocation of NF-kappa B and HIF1alpha in THP-1cells. *Biochem. Biophys. Res. Commun.* 495, 2282-2288. 10.1016/j.bbrc.2017.12.084 29258824

[BIO058852C3] Basile, D. P., Bonventre, J. V., Mehta, R., Nangaku, M., Unwin, R., Rosner, M. H., Kellum, J. A., Ronco, C. and Group, A. X. W. (2016). Progression after AKI: understanding maladaptive repair processes to predict and identify therapeutic treatments. *J. Am. Soc. Nephrol.* 27, 687-697. 10.1681/ASN.2015030309 26519085 PMC4769207

[BIO058852C4] Batkulwar, K. B., Bansode, S. B., Patil, G. V., Godbole, R. K., Kazi, R. S., Chinnathambi, S., Shanmugam, D. and Kulkarni, M. J. (2015). Investigation of phosphoproteome in RAGE signaling. *Proteomics* 15, 245-259. 10.1002/pmic.201400169 25315903

[BIO058852C5] Blondonnet, R., Audard, J., Belville, C., Clairefond, G., Lutz, J., Bouvier, D., Roszyk, L., Gross, C., Lavergne, M., Fournet, M. et al. (2017). RAGE inhibition reduces acute lung injury in mice. *Sci. Rep.* 7, 7208. 10.1038/s41598-017-07638-2 28775380 PMC5543147

[BIO058852C6] Bongarzone, S., Savickas, V., Luzi, F. and Gee, A. D. (2017). Targeting the receptor for advanced glycation endproducts (RAGE): A medicinal chemistry perspective. *J. Med. Chem.* 60, 7213-7232. 10.1021/acs.jmedchem.7b00058 28482155 PMC5601361

[BIO058852C7] Chen, Q., Guan, X., Zuo, X., Wang, J. and Yin, W. (2016). The role of high mobility group box 1 (HMGB1) in the pathogenesis of kidney diseases. *Acta Pharm Sin B* 6, 183-188. 10.1016/j.apsb.2016.02.004 27175328 PMC4856949

[BIO058852C8] D'Agati, V. and Schmidt, A. M. (2010). RAGE and the pathogenesis of chronic kidney disease. *Nat. Rev. Nephrol.* 6, 352-360. 10.1038/nrneph.2010.54 20421886

[BIO058852C9] Eltzschig, H. K. and Eckle, T. (2011). Ischemia and reperfusion--from mechanism to translation. *Nat. Med.* 17, 1391-1401. 10.1038/nm.2507 22064429 PMC3886192

[BIO058852C10] Gasparitsch, M., Arndt, A. K., Pawlitschek, F., Oberle, S., Keller, U., Kasper, M., Bierhaus, A., Schaefer, F., Weber, L. T. and Lange-Sperandio, B. (2013). RAGE-mediated interstitial fibrosis in neonatal obstructive nephropathy is independent of NF-kappaB activation. *Kidney Int.* 84, 911-919. 10.1038/ki.2013.171 23677242

[BIO058852C11] Harashima, A., Yamamoto, Y., Cheng, C., Tsuneyama, K., Myint, K. M., Takeuchi, A., Yoshimura, K., Li, H., Watanabe, T., Takasawa, S. et al. (2006). Identification of mouse orthologue of endogenous secretory receptor for advanced glycation end-products: structure, function and expression. *Biochem. J.* 396, 109-115. 10.1042/BJ20051573 16503878 PMC1450004

[BIO058852C12] Iwata, Y., Furuichi, K., Kitagawa, K., Hara, A., Okumura, T., Kokubo, S., Shimizu, K., Sakai, N., Sagara, A., Kurokawa, Y. et al. (2010). Involvement of CD11b+ GR-1 low cells in autoimmune disorder in MRL-Fas lpr mouse. *Clin. Exp. Nephrol.* 14, 411-417. 10.1007/s10157-010-0309-9 20652350

[BIO058852C13] Iwata, Y., Bostrom, E. A., Menke, J., Rabacal, W. A., Morel, L., Wada, T. and Kelley, V. R. (2012). Aberrant macrophages mediate defective kidney repair that triggers nephritis in lupus-susceptible mice. *J. Immunol.* 188, 4568-4580. 10.4049/jimmunol.1102154 22467656 PMC3340928

[BIO058852C14] Iwata, Y., Satou, K., Tsuzuku, H., Furuichi, K., Senda, Y., Sakai-Takemori, Y., Wada, T., Fujita, S., Miyake, T., Yasuda, H. et al. (2017). Down-regulation of the two-component system and cell-wall biosynthesis-related genes was associated with the reversion to daptomycin susceptibility in daptomycin non-susceptible methicillin-resistant Staphylococcus aureus. *Eur. J. Clin. Microbiol. Infect. Dis.* 36, 1839-1845. 10.1007/s10096-017-2999-3 28477235

[BIO058852C15] Jules, J., Maiguel, D. and Hudson, B. I. (2013). Alternative splicing of the RAGE cytoplasmic domain regulates cell signaling and function. *PLoS ONE* 8, e78267. 10.1371/journal.pone.0078267 24260107 PMC3832623

[BIO058852C16] Kalea, A. Z., Schmidt, A. M. and Hudson, B. I. (2009). RAGE: a novel biological and genetic marker for vascular disease. *Clin. Sci. (Lond)* 116, 621-637. 10.1042/CS20080494 19275767

[BIO058852C17] Kalousova, M., Hodkova, M., Kazderova, M., Fialova, J., Tesar, V., Dusilova-Sulkova, S. and Zima, T. (2006). Soluble receptor for advanced glycation end products in patients with decreased renal function. *Am. J. Kidney Dis.* 47, 406-411. 10.1053/j.ajkd.2005.12.028 16490618

[BIO058852C18] Krakower, C. A. and Greenspon, S. A. (1951). Localization of the nephrotoxic antigen within the isolated renal glomerulus. *AMA Arch. Pathol.* 51, 629-639. 14829136

[BIO058852C19] Lee, E. J., Park, E. Y., Mun, H., Chang, E., Ko, J. Y., Kim, D. Y. and Park, J. H. (2015). Soluble receptor for advanced glycation end products inhibits disease progression in autosomal dominant polycystic kidney disease by down-regulating cell proliferation. *FASEB J.* 29, 3506-3514. 10.1096/fj.15-272302 25934702

[BIO058852C20] Liu, A., Zhang, W., Wang, S., Wang, Y. and Hong, J. (2020). HMGB-1/RAGE signaling inhibition by dioscin attenuates hippocampal neuron damage induced by oxygen-glucose deprivation/reperfusion. *Exp. Ther. Med.* 20, 231. 10.3892/etm.2020.9361 33149785 PMC7604738

[BIO058852C21] Myint, K. M., Yamamoto, Y., Doi, T., Kato, I., Harashima, A., Yonekura, H., Watanabe, T., Shinohara, H., Takeuchi, M., Tsuneyama, K. et al. (2006). RAGE control of diabetic nephropathy in a mouse model: effects of RAGE gene disruption and administration of low-molecular weight heparin. *Diabetes* 55, 2510-2522. 10.2337/db06-0221 16936199

[BIO058852C22] Park, L., Raman, K. G., Lee, K. J., Lu, Y., Ferran, L. J., Jr, Chow, W. S., Stern, D. and Schmidt, A. M. (1998). Suppression of accelerated diabetic atherosclerosis by the soluble receptor for advanced glycation endproducts. *Nat. Med.* 4, 1025-1031. 10.1038/2012 9734395

[BIO058852C23] Prasad, K. (2019). Is there any evidence that AGE/sRAGE is a universal biomarker/risk marker for diseases? *Mol. Cell. Biochem.* 451, 139-144. 10.1007/s11010-018-3400-2 29961210

[BIO058852C24] Sakai, N., Chun, J., Duffield, J. S., Wada, T., Luster, A. D. and Tager, A. M. (2013). LPA1-induced cytoskeleton reorganization drives fibrosis through CTGF-dependent fibroblast proliferation. *FASEB J.* 27, 1830-1846. 10.1096/fj.12-219378 23322166 PMC3633809

[BIO058852C25] Schmidt, A. M., Yan, S. D., Yan, S. F. and Stern, D. M. (2001). The multiligand receptor RAGE as a progression factor amplifying immune and inflammatory responses. *J. Clin. Invest.* 108, 949-955. 10.1172/JCI200114002 11581294 PMC200958

[BIO058852C26] Schmidt, A. M. (2015). Soluble RAGEs - Prospects for treating & tracking metabolic and inflammatory disease. *Vascul. Pharmacol.* 72, 1-8. 10.1016/j.vph.2015.06.011 26130225 PMC4547874

[BIO058852C27] Sharma, A. K., LaPar, D. J., Stone, M. L., Zhao, Y., Kron, I. L. and Laubach, V. E. (2013). Receptor for advanced glycation end products (RAGE) on iNKT cells mediates lung ischemia-reperfusion injury. *Am. J. Transplant.* 13, 2255-2267. 10.1111/ajt.12368 23865790 PMC3776006

[BIO058852C28] Sims, G. P., Rowe, D. C., Rietdijk, S. T., Herbst, R. and Coyle, A. J. (2010). HMGB1 and RAGE in inflammation and cancer. *Annu. Rev. Immunol.* 28, 367-388. 10.1146/annurev.immunol.021908.132603 20192808

[BIO058852C29] Sugihara, T., Munesue, S., Yamamoto, Y., Sakurai, S., Akhter, N., Kitamura, Y., Shiba, K., Watanabe, T., Yonekura, H., Hayashi, Y. et al. (2012). Endogenous secretory receptor for advanced glycation end-products inhibits amyloid-beta1-42 uptake into mouse brain. *J. Alzheimers Dis.* 28, 709-720. 10.3233/JAD-2011-110776 22064071

[BIO058852C30] Wada, T., Yokoyama, H., Furuichi, K., Kobayashi, K. I., Harada, K., Naruto, M., Su, S. B., Akiyama, M., Mukaida, N. and Matsushima, K. (1996). Intervention of crescentic glomerulonephritis by antibodies to monocyte chemotactic and activating factor (MCAF/MCP-1). *FASEB J.* 10, 1418-1425. 10.1096/fasebj.10.12.8903512 8903512

[BIO058852C31] Wang, X., Wang, J., Tu, T., Iyan, Z., Mungun, D., Yang, Z. and Guo, Y. (2018). Remote ischemic postconditioning protects against myocardial ischemia-reperfusion injury by inhibition of the RAGE-HMGB1 pathway. *Biomed. Res. Int.* 2018, 4565630. 10.1155/2018/4565630 29789792 PMC5896327

[BIO058852C32] Wu, H., Ma, J., Wang, P., Corpuz, T. M., Panchapakesan, U., Wyburn, K. R. and Chadban, S. J. (2010). HMGB1 contributes to kidney ischemia reperfusion injury. *J. Am. Soc. Nephrol.* 21, 1878-1890. 10.1681/ASN.2009101048 20847143 PMC3014003

[BIO058852C33] Wuthrich, R. P., Glimcher, L. H., Yui, M. A., Jevnikar, A. M., Dumas, S. E. and Kelley, V. E. (1990). MHC class II, antigen presentation and tumor necrosis factor in renal tubular epithelial cells. *Kidney Int.* 37, 783-792. 10.1038/ki.1990.46 2407890

[BIO058852C34] Yamamoto, Y., Harashima, A., Saito, H., Tsuneyama, K., Munesue, S., Motoyoshi, S., Han, D., Watanabe, T., Asano, M., Takasawa, S. et al. (2011). Septic shock is associated with receptor for advanced glycation end products ligation of LPS. *J. Immunol.* 186, 3248-3257. 10.4049/jimmunol.1002253 21270403

[BIO058852C35] Yonekura, H., Yamamoto, Y., Sakurai, S., Petrova, R. G., Abedin, M. J., Li, H., Yasui, K., Takeuchi, M., Makita, Z., Takasawa, S. et al. (2003). Novel splice variants of the receptor for advanced glycation end-products expressed in human vascular endothelial cells and pericytes, and their putative roles in diabetes-induced vascular injury. *Biochem. J.* 370, 1097-1109. 10.1042/bj20021371 12495433 PMC1223244

[BIO058852C36] Zhang, C., Dong, H., Chen, F., Wang, Y., Ma, J. and Wang, G. (2019). The HMGB1-RAGE/TLR-TNF-alpha signaling pathway may contribute to kidney injury induced by hypoxia. *Exp. Ther. Med.* 17, 17-26. 10.3892/etm.2018.6932 30651760 PMC6307518

[BIO058852C37] Zhu, C., Liu, Y., Guan, Z., Zhou, Y., Liu, F. and Zhang, T. (2018). Hypoxia-reoxygenation induced necroptosis in cultured rat renal tubular epithelial cell line. *Iran J. Basic Med. Sci.* 21, 863-868. 30186575 10.22038/IJBMS.2018.26276.6444 PMC6118090

